# Effects of chemotherapy and immunotherapy on microbial diversity in TME and engineered bacterial-mediated tumor therapy

**DOI:** 10.3389/fimmu.2023.1084926

**Published:** 2023-02-02

**Authors:** Heng Zheng, Xianxian Chen, Qiyang Li, Yuqi Liu, Jinzhong Cai

**Affiliations:** ^1^ School of Chemistry, Chemical Engineering and Life Sciences, Wuhan University of Technology, Wuhan, China; ^2^ School of Resources and Environmental Engineering, Wuhan University of Technology, Wuhan, China; ^3^ Department of Interventional Radiology, Shenzhen People’s Hospital (The First Affiliated Hospital, Southern University of Science and Technology), Shenzhen, China

**Keywords:** tumor, microbiome, chemotherapy, immunotherapy, engineered bacteria

## Abstract

Tumor microbiota is a group of microorganisms located in tumor tissues with rich diversity that can promote tumorigenesis and development, and different types of tumors have different tumor microbiotas, which has important implications for tumor research, detection, and clinical treatment. In this review, we examine the diversity of the tumor microbiota, discuss the impact of chemotherapy and immunotherapy on tumor microbiota diversity, and summarize recent advances in the use of genetically engineered bacteria for the treatment of tumors. In addition, we propose key questions that need to be further addressed by the tumor microbiota.

## 1 Introduction

The cancer is a major global health issue. In the past, tumorigenesis and microbial infection were considered as independent diseases from each other, since the discovery of bacteria in tumor tissues, it has revolutionized the perception of the relationship between microorganisms and tumors. Microorganisms exist in the tumor microenvironment (TME), which are located inside tumor tissues or in tumor cells, and they differ between healthy populations and cancer patients. The relationship between the microorganisms and the tumor cells (or other parts of the TME) is most likely a symbiotic relationship, rather than a simple positional one. Tumors can create more suitable conditions for microorganisms to survive and reshape the microbial spectrum, and at the same time, microorganisms can also promote tumorigenesis and progression by establishing an inflammatory environment and influencing host immunity(1). Fu et al. showed that bacteria within breast tumor cells contribute to cancer cell metastasis, in part by enhancing tumor cell survival in response to mechanical fluid shear stress(2). Prostate cancer is a malignant tumor and its occurrence and progression are influenced by a variety of factors, including ethnicity, and a growing number of studies have confirmed that the prostate microbiota(it may come from urinary tract flora and intestinal flora) may play an important role in the occurrence, progression, and prognosis of prostate cancer, and that microorganisms and their metabolites influence the occurrence and metastasis of cancer cells (3). In addition, there is a close correlation between some systemic diseases and microbiota. For example, patients with oral cancer had a more enriched oral microbiota composition in precancerous lesions, and at the genus level, the main differentially enriched taxa were *Prevotella*, and *Carnobacterium*, *Peptostreptococcus*(P<0.05)(4). Gut microbiota plays an important role in colorectal carcinogenesis, and *Bacteroides fragilis*, *Helicobacter pylori*, *Peptostreptococcus anaerobius*, Sulfate-reducing bacteria, and *Fusobacterium nucleatum* can mediate cancer development (5).

The microorganisms present in the TME (e.g. viruses, bacteria, fungi and protozoa), regulate the TME in a species-dependent manner due to the specific physiological and pathological characteristics of each microorganism, thereby inhibiting or promoting tumor growth (6). Recent studies of cancer-associated microbial communities have enriched the body of knowledge on the interactions between microorganisms, the immune system, and tumor cells.

Microbial communities that have an impact on tumor progression and are associated with tumors are defined as the tumor microbiota, but this article focuses on microbes between tumor tissues. For example, studies of bacteria in the induction of lung cancer have been largely neglected, and although there is a correlation between lung cancer and the lung microbiota, and the effect of microbes on lung cancer progression has been much confirmed, little is known about the underlying mechanisms. Microorganisms can promote tumorigenesis and progression through the production of toxins(such as Cytolethal distending toxin, cytotoxic necrotizing factor 1, and *Bacteroides fragilis* toxin) and other pro-inflammatory factors(7). The human microbiome is composed of different microorganisms that play important roles in processes such as metabolism and host defense, and high-throughput sequencing technologies can contribute to a broader understanding of microbial composition and diversity. Through high-throughput sequencing, microorganisms in TME can be comprehensively identified, and in the future, these microorganisms can be used as effective probes, monitors, drugs. Therefore, understanding the tumor microbiota is important for clinical care and research of tumors.

Emerging evidence suggests many roles for the microbiota in tumors, including immune responses to tumors, Effects to the TME, and alterations of the microbiota by chemotherapy. The balance of the microbiota in normal tissues helps to defend against tissue lesions, microbiota in TME will influence tumor development and treatment, and a comprehensive understanding of the tumor microbiota will improve the way cancer patients use anticancer drugs. Although the relationship between the microbiota and cancer has made significant progress in the past, however, the relationship between bacteria within tumors and cancer is unclear. A growing body of evidence suggests that intratumoral bacteria are an important component of the cancer microenvironment and that they may directly influence tumorigenesis, progression, and treatment, as well as modulate innate and adaptive immunity to potentially influence tumor immunotherapy(8). Furthermore, the interaction of the tumor microbiota with TME has recently been mimicked, making related microbes potentially powerful cancer therapeutic agents. Bacteria have been used as anticancer agents since the 19th century, when William B. Coley used *Streptococcus pyogenes* to successfully heal patients with inoperable sarcomas(9). With the development of synthetic biology, several bacteria have been genetically engineered and their applicability for cancer treatment has increased significantly. Compared to other therapeutic approaches, genetically engineered bacteria for cancer treatment have unique properties in that they can specifically accumulate within tumors and inhibit cancer growth(10). In addition, genetically engineered bacteria can be combined with materials, used as carriers for delivering antitumor drugs, or used in combination with radiotherapy to improve the effectiveness of cancer treatment.

## 2 Microbial diversity in TME

Cancer is a series of genetic changes that transform normal cells into tumor cells, caused by biological, chemical or physical factors, including the microbiota. The link between microbial species and cancer is mainly stimulated by immune responses in tumor cells, such as inflammation induction. The association between bacteria and cancer has recently been found to be through two mechanisms, the first stimulating chronic inflammation and the second producing oncogenic metabolites (11). Conventional cancer treatments include chemotherapy, radiotherapy, surgery, and immunotherapy, which increase the chances of survival of patients, but these therapies are associated with many side effects, including high toxicity to tissues and normal cells.

Poore et al. (12) analyzed nucleic acid sequences from human cancers, adjacent tissue samples and blood samples to reveal the microorganisms present in various tumors. They used The Cancer Genome Atlas (TCGA) to analyze data from 33 cancer types, with a total of more than 17,000 samples from about 10,000 patients, and they used multiple computational methods, including independently trained artificial intelligence models, to filter, normalize, and classify microbial sequences in these samples, resulting in findings that approximately one-third mapped to known bacterial, archaeal, or viral sequences. By training machine learning models, the above microbial sequences can be used to differentiate cancer types, and cancerous versus normal tissue. In addition, *Clostridium perfringens* was found in gastrointestinal tumors, and viruses such as Metapneumovirus and Hepatovirus were found in cervical, head and neck, and liver cancers. Poore et al. also analyzed the characteristics of microorganisms in the blood of cancer patients, and showed that blood-borne microbial DNA can be used to distinguish between cancer types. The tumor microbiota can be classified according to the habitat of microorganisms in TME, which can be divided into commensal microbial enrichment (esophagus, oral cavity, colon, etc.) and acquired microbial enrichment (lung, liver, breast, etc.). The microbial diversity in different tumors is shown in **Table 1**.

**Table 1:** Statistical table of microbial diversity of different tumor types.

Cancer Type	Microbial diversity	Reference
Breast tumors	*Pseudomonas* sp., *Brevundimonas*, *Staphylococcus*, *Pseudomonas aeruginosa*	13
Pancreatic ductal adenocarcinoma	Enterobacteriaceae	14
Esophageal squamous cell carcinoma	Proteobacteria, Firmicutes, Bacteroidetes, *Deinococcus-Thermus*, *Actinobacteria*, *Streptococcus*, *Labrys*; *Rhodotorula mucilaginosa*, *Porphyromonas endodontalis*, *Neisseria subflava*, *H.pylori*, *Acinetobacter parahaemolyticus*, *Acinetobacter rhizosphaerae*	15, 16
Colorectal cancer	*Actinomyces*, *Schaalia cardiffensis*, Bacteroidia, Gammaproteobacteria, *Pseudomonas*, *Actinomycetes*; Firmicutes, Bacteroidetes, Proteobacteria	17, 18
Pancreatic cancer	*Pseudoxanthomonas*, *Streptomyces*, *Saccharopolyspora*, *Bacillus clausii*	19
Lung cancer	*Veillonella*, *Megasphaera* sp.; *Modestobacter*, *Propionibacterium*, *Enterobacteriaceae*	20, 21
Hepatocellular carcinoma	Bacteroidetes, Firmicutes, Proteobacteria, *Bacteroides*, *Romboutsia* and uncultured bacterium of *Lachnospiraceae*	22
Carcinoma of the anus	*Clostridia*, *Clostridiales*, *Actinobacteria*	23

### 2.1 Congenital microbial enrichment

Esophageal squamous cell carcinoma(ESCC) is a disease of high malignancy. Shen et al. (15) analyzed the microbial community structure and differences in tumor tissues and adjacent non-tumor tissues of 51 patients with esophageal squamous cell carcinoma by 16S rDNA sequencing and showed that Proteobacteria, Firmicutes, Bacteroidetes, *Deinococcus-Thermus* and *Actinobacteria* were the predominant bacteria in tumor tissues and adjacent non-tumor tissues. At the genus level, *Streptococcus*(median relative abundance of 6.39%) and *Labrys*(median relative abundance of 11.1%) were the bacteria with the highest relative proportions in tumor and adjacent non-tumor tissues. *Labrys* mostly exists in the natural environment, and its high abundance might be a contamination or an artifact. In addition, the complexity of microbial interactions in tumor tissues was weaker than in adjacent non-tumor tissues, with 24 taxa of microorganisms statistically different between tumor and adjacent non-tumor tissues, and lipopolysaccharides were more significant in the synthesis and metabolism of the tumor tissue microbiota. Using the same method, Yang et al. investigated the esophageal microbiota of 18 ESCC patients and 11 physiologically normal (PN) esophageal patients and found that the composition of the microbiota in the tumor tissues of ESCC patients was significantly different from that of PN patients. The ESCC microbiota was characterized by reduced microbial diversity and decreased abundance of Bacteroidetes, Fusobacteria and Spirochetes. In addition, the inclusion of these taxa in the microbial dysbiosis index distinguished well between the ESCC and PN groups. Functional analysis showed that the nitrate reductase and nitrite reductase functions were altered in the ESCC microbiota compared to the PN group, for reasons that need to be further investigated (24). In addition, esophageal microbiota may influence the pathophysiology of ESCC. 16S rRNA sequencing of samples from esophageal tumor tissues and tumor-adjacent tissues of 120 ESCC patients resulted in the detection of 56 taxa with different abundances, such as *Rhodotorula mucilaginosa*, *Porphyromonas endodontalis*, *Neisseria subflava*, *H.pylori*, *Acinetobacter parahaemolyticus*, and *Acinetobacter rhizosphaerae.* Microbial diversity and composition of ESCC tumor tissues were significantly different from tumor-adjacent tissues, and this inter-subjective beta-diversity variation was mainly caused by region and sampling season. Quantitative PCR showed a higher species abundance of *P.endodontalis* and a lower abundance of *H.pylori* in tumor-adjacent tissues. In addition, denser and more complex association networks were formed in tumor-adjacent tissues than in tumor-adjacent tissues. In addition, alterations in microbial symbiotic networks and functional pathways in ESCC tissues may be involved in the oncogenic process of the local microenvironment of ESCC (16).

In addition to lung cancer, the third most common form of cancer is colorectal cancer (CRC), and the incidence of sporadic young adult colorectal cancer (yCRC) has been increasing. Xu et al. (18) found microbiota differences between yCRC patients and patients with old-onset colorectal cancer(oCRC). 16S rRNA sequencing analysis revealed decreased tumor microbial α diversity in yCRC, suggesting the presence of microbial community dysbiosis in yCRC. At the genus level, two microbiota were enriched in yCRC patients compared to the oCRC group: *Enterococci* and *Castellaniella*. *Castellaniella* mostly exists in the intestinal environment, and its high concentration might be a contamination or an artifact. Among all yCRC samples, Proteobacteria being the most abundant phylum, while *Actinomyces* and *Schaalia cardiffensis* were the key microbiota in the yCRC group. Correlation analysis revealed that *Actinomyces* coexisted with various tumor-promoting microbial taxa, including *Bacteroidia*, *Gammaproteobacteria* and *Pseudomonas.* This is because *Actinomycetes* in yCRC co-localized with cancer-associated fibroblasts and activated the TLR2/NF-kappa B pathway, reducing CD8+T lymphocyte infiltration in the yCRC microenvironment. The tumor microbiota plays an important role in promoting tumorigenesis and thus has the potential to be a non-invasive tool and target of intervention for antitumor therapy. Although the relevance of the gut microbiota to checkpoint immunotherapy has been thoroughly explored, there are few data on the local tumor microbiota. Boesch et al. used 16S rRNA gene amplicon sequencing to characterize the molecular profile of patients treated with PD-1/PD-L1-targeted checkpoint inhibitors. The results showed a significant diversity of tumor microbiota, with high proportions of Firmicutes, Bacteroidetes and Proteobacteria. Combined with clinical data, high microbial diversity was associated with improved patient survival. Furthermore, the presence of γ-proteobacteria was associated with low PD-L1 expression and poor response to checkpoint-based immunotherapy, which translated into poor survival. Thus, new microbiota-specific/derived biomarkers could be used for the prediction and prognosis of immune checkpoint therapies (17).

Which microorganisms are functionally active in the endometrium of patients with endometrial cancer (EC) and how the human host responds to functionally active microorganisms? More than 5,000 functionally active microorganisms were described by metatranscriptomic analysis in 9 endometrial cancer patients and 8 normal individuals, and differences in microorganisms between the EC and normal groups were analyzed. The results revealed that these microorganisms are involved in some of the metabolic processes of endometrial cancer, such as 6-sulfosalicylic acid Lewis x epitope, and N-acetyl-β-glucosaminyl. In addition, the host-microbiota crosstalk in endometrial cancer includes many biological processes, mainly functions related to tumor migration and Apelin signaling pathway. *Pannonibacter phragmitetus* with high abundance in EC endometrium was significantly and positively correlated with Wnt signaling pathway, IL-17 signaling pathway and MAPK signaling pathway. In conclusion, functionally active microorganisms in EC endometrium play a crucial role in tumorigenesis and migration, and their role in the treatment of endometrial cancer cannot be ignored (25).

### 2.2 Acquired microbial enrichment

One-fifth of cancers are attributed to infectious agents, usually viruses, followed by parasites and bacteria (26). There is a growing number of evidence that changes in microbial communities influence cancer development as well as treatment. By analyzing microbial sequences from head and neck squamous cell carcinoma (HNSC), hepatocellular carcinoma of the liver (LIHC), and gastric adenocarcinoma (STAD), bacterial diversity within cancer samples was found to be related with the survival rate of associated cancers, and varies by tissue type and is dependent on interactions with gender and race (27). Patients with pancreatic cancer (PDAC) resection survive for less than 5 years, but a small percentage survive longer, by dissecting the role of PDAC tumor microbiota and immune system in influencing long-term survival. Using 16S rRNA sequencing, the tumor microbiota of PDAC patients with short-term survival and long-term survival were analyzed, and the results showed that long-term patients have a higher alpha diversity in their tumor microbiota, and that *Pseudoxanthomonas*-*Streptomyces*-*Saccharopolyspora*-*Bacillus clausii* can be used as a tumor microbiota signature that is highly predictive of long-term survival. Immediately following the human-mouse fecal microbiota transplantation experiment, it was found that cross-linking of the PDAC microbiota with the gut microbiota influences the host immune response and disease (19).

Lung cancer is one of the most common cancers and the impact of the bacterial flora in the lung microenvironment on lung cancer development and progression is not yet clear. The pathogenesis and progression of lung cancer are associated with increased respiratory bacterial loads and changes in bacterial communities, due to the fact that the microbiota affects tumors in multiple ways, including carcinogenesis, metastasis, angiogenesis, and therapy. The microbiota may enhance tumor susceptibility by altering metabolic, promoting inflammation, and facilitating immune responses. The microbiota(such as *Haemophilus influenzae*, *Morganella morganii*, and *Escherichia fergusonii*) may also modulate tumor metastasis by altering multiple cellular signaling pathways and participate in tumor angiogenesis through vascular endothelial growth factor (VEGF), endothelial cells (EC), inflammatory factors and inflammatory cells (28). Tumor angiogenesis not only maintains tumor growth at the primary site, but also promotes tumor metastasis. Therefore, angiogenesis is an important mediator of microbiota-tumor interactions. Antibiotic-induced microbiota alterations can regulate tumor growth and metastasis. In addition, the microbiota influences the efficacy and toxicity of tumor immunotherapy and chemotherapy. Mao et al.(29) investigated microbial composition and diversity in tumor tissues and adjacent tissues of 55 lung cancer patients and showed that tumor samples had significantly lower alpha diversity, higher taxon interactions, and fewer potentially pro-inflammatory microbial genera compared to non-malignant adjacent tissues, but no significant differences in overall microbiota heterogeneity. It should be noted that sometimes lung cancer may not be driven by the infection itself, but by a major transformation of its microbial community. Lung microbiota diversity is an important indicator of malignant transformation in lung cancer, with alpha diversity tending to be lower in lung cancer patients and conversely beta diversity not significantly different in the lungs of healthy and cancer patients (30). Therefore, the microbiota should be considered an important diagnostic and preventive indicator of lung cancer. Lee and colleagues analyzed the differences in the microbiotas of patients with benign and malignant lung tumors by 16S rRNA high-throughput sequencing and suggested that *Veillonella* and *Megasphaera* spp. could be used as biomarkers for lung cancer (20). In addition, compared to adjacent normal tissues, lung cancer tissues contained higher levels of *Modestobacter* but lower levels of *Propionibacterium* and *Enterobacteriaceae* (21). In terms of diversity, there was no significant difference in beta diversity between non-malignant lung cancer tissues and lung tumor tissues, but alpha diversity of the microbiota was significantly reduced in lung tumor tissues (30). During carcinogenesis, microecological deviations from homeostasis gradually create a microenvironment that promotes cancer induction and ultimately causes carcinogenesis. Thus, microecological imbalances in the lung may affect lung carcinogenesis through inflammation, immunity, metabolism and genotoxicity.

Hepatocellular carcinoma is the fourth leading cause of cancer-related death, and hepatocellular carcinoma is a primary liver cancer resulting from chronic hepatitis caused by multiple susceptibility factors such as viruses, alcohol consumption, and nonalcoholic fatty liver disease. Komiyama et al. characterized the tumor-associated microbiota in patients with hepatocellular carcinoma. It was found that the number of amplicon sequence variants in the tumor-associated microbiota was significantly increased compared with non-tumor areas of the liver, and the tumor-associated microbiota consisted mainly of Bacteroidetes, Firmicutes, and Proteobacteria. In addition, *Bacteroides*, *Romboutsia* and uncultured bacterium of Lachnospiraceae could be used as signature taxa for primary liver cancer (22).

In addition to clinical samples, Zhang et al. (31) used high-throughput sequencing to identify and compare microbial diversity in human and murine tumors, and principal component analysis and β-diversity showed low microbial similarity between mouse artificial tumors, mouse spontaneous tumors, and human tumors, with *Serratia*, Pseudomonas spp. and *Ochrobactrum* as the dominant bacteria at the genus level. The intracellular microbiota of tumors is an emerging class of tumor components whose biological functions are still unclear. Fu et al. (32) used a mouse model of spontaneous mammary tumors to explore the function of these intratumoral bacteria. It was found that depletion of intratumoral bacteria significantly reduced metastasis of tumor cells without affecting the growth of the primary tumor. Furthermore, during metastatic colonization, intratumoral bacteria enhanced resistance to fluid shear stress by reorganizing the actin cytoskeleton, thereby promoting host cell survival. In addition, bacterial strains isolated from tumor cells can promote tumor metastasis. Although small in number, microorganisms in tumor cells play an important role in promoting cancer metastasis, and there is a need to intervene to advance tumor control.

## 3 Effect of chemotherapy on microbial diversity in TME

The microbiota in TME may also alter the efficacy and toxicity of chemotherapy through the effects of chemotherapeutic drug metabolism, enzymatic degradation, etc., thus reducing diversity and variability (33). *In vitro* cell line experiments, the efficacy of chemotherapeutic drugs(gemcitabine) on cancer cell killing seems to be influenced by different species of bacteria, some improving cell killing and some reducing efficacy (34). The specific bacterial species and the mechanism of influence need to be further explored. To address this issue of microbial metabolism of anticancer drugs in the TME leading to chemotherapy failure, Qiu et al. (35) developed antibiotic and anticancer drug co-delivery micelles, called colistin cross-linked gemcitabine micelles (CCGM), capable of on-demand release without compromising their antimicrobial and anticancer effects. Once the CCGM is delivered to the TME, intracellular glutathione triggers the release of colistin and gemcitabine to inhibit the growth of microorganisms in the tumor, thereby eliminating microbial-induced tumor drug resistance. The combinations and ratios of different antibiotics and anticancer drugs should be further explored. In addition, antibiotics inhibit the toxicity of oxaliplatin to cells in tumors by reducing microbiota-dependent ROS production in TME (36).

Breast tumors have their specific microbiota, which is different from normal breast tissue. By aseptically collecting breast tumor tissues from patients receiving neoadjuvant chemotherapy and patients who had not received treatment at the time of surgery and using 16S rRNA high-throughput sequencing, it was found that the number of *Pseudomonas* spp. in breast tumors increased significantly after chemotherapy administration (doxorubicin), and primary breast tumors from patients with distant metastases showed increased abundance of *Brevundimonas* and *Staphylococcus* spp. and the presence of *Pseudomonas aeruginosa* in breast tumor tissue was confirmed by IHC staining (13). In conclusion, chemotherapy alters the tumor microbiota of breast tissue and specific microorganisms are associated with tumor recurrence. Future studies could increase the size of the patient populations to gain insight into the role of microbiota in chemotherapy and develop novel bacterial biomarkers that can predict distant tumor metastasis.

The development, treatment and post-treatment survival of pancreatic ductal adenocarcinoma (PDAC) are associated with the tumor microbiota. Tumor tissue and normal pancreatic tissue samples were obtained from 27 patients who had undergone surgical resection of PDAC, and 16S rRNA high-throughput sequencing showed detectable bacterial communities in 26 of the 54 samples. The relative abundance(P = 0.04) of microbiota from Enterobacteriaceae was significantly higher in samples from patients who underwent biliary stenting or neoadjuvant therapy with a combination of gemcitabine and paclitaxel. The above findings suggest that biliary stenting and neoadjuvant chemotherapy are associated with alterations in tumor microbiota diversity that promote infiltration and growth of intra-tumor bacteria in PDAC (14). Further exploration of whether specific bacterial communities contribute to increased chemoresistance can follow, which is critical for optimizing future therapies.

Pancreatic cancer is a malignant tumor of the digestive system with a very high mortality rate. Gemcitabine-based chemotherapy is the main treatment for end-stage pancreatic cancer, but its therapeutic effect is often poor. Microorganisms(*Escherichia-Shigella*, *Malassezia* spp., *Adenoviruses*, *Herpes simplex* virus type 1, *Vaccinia* viruses) in TME not only play an indirect role in the development and progression of pancreatic cancer, but also influence the effectiveness of chemotherapy to some extent. In addition, microorganisms may be important biomarkers for predicting pancreatic carcinogenesis and detecting pancreatic cancer prognosis. However, the available data are insufficient, for example, what are the mechanisms by which lysoviruses and bacteria affect pancreatic cancer chemotherapy? The next step could be focus on specific microbial species in TME to improve the efficacy of chemotherapy in the treatment of pancreatic cancer (37).

Patients with localized squamous cell carcinoma of the anus (SCCA) have few treatment options after experiencing treatment toxicity or recurrence, and the impact of the microbiota in TME on treatment toxicity and its potential use as a predictive biomarker could improve the prognosis of these patients. Lin et al. (23) used 16S rRNA high-throughput sequencing of the tumor site of 22 patients with SCCA to the microbiota and showed that alpha diversity remained relatively stable throughout the chemoradiotherapy period and the tumor microbiota changed during and after chemotherapy. In addition, different levels of bacterial enrichment (P = 0.03 for all), such as *Clostridia*, *Clostridiales*, and *Actinobacteria*, occurred at specific time points (during CRT) during treatment, which would play an important role in predicting the tumor microbiota for SCCA patients.

## 4 Effect of immunotherapy on microbial diversity in TME

As a very promising strategy for oncology treatment, immunotherapy is highly relevant to microorganisms in TME. Immunotherapy has shown great promise in the treatment of patients with metastatic malignancies, dramatically changing the cancer treatment, especially after the discovery of immune checkpoint inhibitors. However, the response to immunotherapy is heterogeneous and often transient. More importantly, a large proportion of cancer patients are resistant to this treatment. Much effort has been expended to identify reliable biomarkers to accurately predict clinical response to immunotherapy (38–40). Unfortunately, such biomarkers are still lacking and the mechanisms underlying their efficacy and safety are poorly understood. Emerging evidence suggests that microbes in TME can alter the efficacy and toxicity of immunotherapies by modulating the local and systemic immune response of the host. Therefore, it is critical to use the microbiota to develop biomarkers that can be used for cancer immunotherapy (41). A growing number of findings demonstrate that microorganisms in TME have a significant impact on tumorigenesis, progression and metastasis. In particular, the effect of bacteria on tumor immunity. Bacteria can suppress the function of the immune system through a variety of mechanisms, and some bacteria(such as *E. coli*, *Salmonella*, *Clostridium butyricum*, *Listeria*) can enhance immunity and inhibit tumor progression (42).

Intratumoral and tumor local microbes remain a promising direction for oncology research. Boesch et al. (17) found that lung tissue from patients with non-small cell lung cancer had a higher abundance of Gammaproteobacteria compared to healthy lung tissue, which was associated with lower expression of programmed death ligand 1 and worse overall survival under immune checkpoint inhibitor(ICI) therapy. Furthermore, an interesting study showed that bacteria(*Akkermansia muciniphila*) within mouse PDAC tissue can drive IL-33 secretion, which further recruites and activates Th2 cells and type 2 innate lymphoid cells in tumor tissue, ultimately leading to suppression of antitumor immune responses and promoting tumor progression (43). Bone marrow cells are a major component of the TME and can induce antitumor immune responses, mainly promoting immune evasion, tumor progression and metastasis. Myeloid cells respond to environmental factors, including signals from commensal microbes. The commensal microbiota is a major player in influencing carcinogenesis, tumor progression, cancer comorbidity, and treatment outcomes by setting inflammatory/immune tone and modulating host responses to oncogenic pathogens, cancer-associated inflammation, and tumor-induced tissue damage (44). In general, oncogenic microbial infections and microbiota in TME dysbiosis are strongly associated with tumorigenesis, and intratumoral and local tumor microbes contribute significantly to tumor progression. However, the causal relationship between them still needs further investigation.

## 5 Bacteria-mediated cancer therapy

Conventional cancer treatments, including radiotherapy, chemotherapy, surgery and immunotherapy, have limitations that negatively affect the health of patients. Bacterial-mediated tumor therapy is emerging as a promising approach in cancer treatment because specialized anaerobic microorganisms (e.g., *Clostridium* spp., *Bifidobacterium* spp.) or pathogenic anaerobic microorganisms (e.g., *Lactobacillus* spp. and *Lactobacillus* sp.) can penetrate and proliferate in tumor hypoxic regions. Anaerobic bacteria are known to cause tumor regression and inhibit metastasis through multiple mechanisms (toxin production, anaerobic lifestyle, and synergy with anticancer drugs) (45). The above-mentioned bacterial functions have the potential to be used as a complementary tool to conventional cancer therapy. In the last decade or so, the use of bacteria as a treatment for cancer has attracted a lot of attention. There are various approaches to the use of bacteria for cancer treatment, including the use of live, attenuated or genetically engineered microorganisms, bacterial-based cancer immunotherapy, bacterial vectors for genetically targeted enzyme precursors, and probiotics. Ma et al. (46) analyzed the composition of prostate cancer intratumoral bacteria to determine the impact of the microbiota on metastatic tumor growth. Specific microorganisms (*Listeria monocytogenes* and *Metacoccus radiodurans* JCM 2831) were identified that could significantly halt prostate cancer progression. Bacterial therapies, either alone or in combination with other therapies, can have a positive impact on cancer treatment. Moreover, bacteria can be used as carriers of drugs, genes or therapies, which is a breakthrough in the treatment of cancer (11). Bacteria-directed multimodal synergistic cancer therapies (e.g., photothermal therapy, targeted synergistic approaches, and bacterial metabolite therapy) offer a range of advantages. Representative studies of bacterial-directed multimodal synergistic cancer therapies are described next.

### 5.1 Photothermal therapy

For the medical application of engineered bacteria, strict regulation of the function of engineered bacteria has been the goal pursued. However, existing regulation methods do not meet the needs of *in vivo* applications of engineered bacteria. Li et al. (47) constructed thermosensitive engineered bacteria that could respond to thermal stimulation within 30 min and could colonize the TME in mice, and thermal stimulation could control the production of protein tumor necrosis factor α by engineered bacteria in tumors, and the growth of tumors was significantly inhibited, indicating that heat can be used as a strategy to precisely control *in vivo* engineered bacteria. Furthermore, intra-tumor hypoxia significantly limits the susceptibility of solid tumors to oxygen-dependent photodynamic therapy(PDT). Ding et al. (48)developed a novel engineered bacterium capable of targeting hypoxic tumor tissues and effectively mediating photodynamic therapy for these tumors. *E.coli* were genetically engineered to express peroxidase, and black phosphorus quantum dots(BPQDs) were attached to the surface of these bacteria by electrostatic adsorption to produce an engineered *Escherichia coli*/BPQDs(EB) system. After intravenous injection, EBs can target hypoxic tumor tissue, and when laser-driven EBs produce reactive oxygen species(ROS) and disrupt the bacteria’s membrane, it is able to release catalase and subsequently degrade hydrogen peroxide to produce oxygen. The increased oxygen alleviates intra-tumor hypoxia, thereby enhancing BPQDs-mediated photodynamic therapy. The system can kill tumor cells effectively *in vivo*, showing good therapeutic efficacy.

### 5.2 Targeted synergistic approach

A biologically targeted synergistic system consisting of genetically engineered bacteria and multifunctional nanoparticles, Wang et al. (49) genetically modified *E.coli* to carry an acoustic reporter gene encoding the formation of gas vesicles (GVs), then targeted the tumor hypoxic environment in mice, followed by ultrasound imaging and collaborative FUAS, and enriched multifunctional nanoparticles in the tumor target area by electrostatic adsorption. Multifunctional cationic lipid nanoparticles containing IR780, perfluorohexane, and banoanthraquinone hydrochloride (AQ4N) were then co-loaded into the tumors for targeted multimodal imaging and enhanced FUAS efficacy. AQ4N was stimulated by the tumor hypoxic environment to kill tumor cells in synergy with focused ultrasound ablation procedures. Natural bacteria are potential targets for cancer therapy due to their unique autonomously driven and hypoxic targeting properties. Genetically engineered bacteria can effectively cross complex physiological barriers to deliver antitumor drugs to deep tumor tissues with a favorable biosafety profile. In addition, bacteria can secrete cytokines and activate antitumor immune responses in the TME, leading to tumor suppression. By combining bacterial-based drugs with other therapeutic approaches, synergistic antitumor strategies can be developed (50). Bacteria-driven drug delivery systems are of high interest for their therapeutic specificity and efficacy in cancer treatment. YB1 is a particularly attractive transgenic safe strain of *Salmonella typhimurium* that can penetrate hypoxic tumor tissue, with self-driven properties, while avoiding damage to normal tissue. Chen et al. (51) have attached nanophotosensitizers (indocyanine (ICG)-loaded nanoparticles INPs) attached to YB1 surface with amide bonds to develop bio/non-biological cross-linked systems (YB1-INPs) for tumor precision therapy. The YB1-INPs therapeutic strategy demonstrated specific hypoxic targeting to solid tumors, thanks to the combined contribution of tumor tissue destruction and nutrient uptake production by bacteria after photothermal treatment, with bioaccumulation of YB1-INPs compared to no photothermal intervention was significantly increased by 14-fold. Furthermore, YB1-INPs were spread throughout large solid tumors and exhibited reliable and efficient photothermal killing ability under near-infrared (NIR) laser irradiation to eradicate large solid tumors without recurrence. This bacterially driven hypoxia-targeted delivery strategy is valuable for large solid tumor treatment with great applications.

### 5.3 Bacterial metabolite therapy

Bacterial-targeted enzyme precursor drug therapy (BDEPT) is an emerging alternative targeted and tumor-specific approach. Microbial-based oncology treatments include lysoviral therapy and bacteriotherapy, both of which use tumor-specific infectious microorganisms to treat cancer. The approach is based on the conversion of non-toxic precursor drugs into toxic drugs in the TME by bacterial enzymes. BDEPT therapy is based on the ability of *E.coli* DH5 to activate glycyrrhizic acid(GL), a naturally occurring non-toxic compound derived from licorice, to glycyrrhetinic acid(GA) only in the TME, and the bacteria can effectively activate GL to GA to inhibit the growth of tumor cells. The anticancer effect of the bacteria used in combination with GL was studied in a mouse model of colon cancer and it was found that the combination treatment greatly inhibited tumor growth and histopathological analysis also showed an increase in apoptosis of tumor cells after the combination treatment with no significant damage to the spleen and liver of the mice (52). Therefore, the BDEPT approach can be considered an effective tumor-specific therapy. l-arginine availability in tumors is a key determinant of effective antitumor T-cell responses, and increased intra-tumor l-arginine concentrations may greatly enhance the antitumor response to immune checkpoint inhibitors. However, there is currently no way to locally increase intra-tumor l-arginine levels, and Canale et al. used a synthetic biology approach to develop an engineered *E. coli strain Nissle 1917*. Colonization of tumors with these bacteria increased intra-tumor L-arginine concentrations, and increased the number of tumor-infiltrating T cells, and had a significant synergistic effect with PD-L1 blocking antibodies in tumor clearance (53). In addition, a novel strategy combining bacterial therapy with high-intensity focused ultrasound (HIFU) therapy may lead to more effective breast cancer treatment. Acoustic reporter genes (ARG) were successfully expressed in *E.coli* by genetic engineering to produce protein nanoparticle-air sacs (GVs). After intravenous injection, GVs-containing *E.coli* can specifically target tumor sites, colonize continuously in the TME, and significantly inhibit tumor growth. Meanwhile, GVs-containing *E.coli* could effectively synergize HIFU treatment both *in vitro* and *in vivo*. The tumor inhibition rate of the combination treatment group could be as high as 87% compared to the control group (54).Therefore, bacterial metabolite therapy has some prospects for application.

## 6 Conclusion

In recent years, oncology treatment has made great progress, which has significantly improved the quality of life and survival rate of cancer patients. However, conventional cancer treatment still has many limitations. For the past few years, with the rise of tumor microbiota and bacterium-based therapies, it is expected to remedy these limitations. For example, bacterial species such as *Salmonella*, *Clostridium* and *Listeria* have been shown to control tumor growth and improve prognosis in experimental animal models and clinical settings (55). The current research and clinical status of the tumor microbiota should be of interest to relevant scientists and clinicians, and future work could be carried out in the following areas.The accuracy of gene sequencing should be enhanced to provide a more comprehensive and accurate understanding of the microbiotas of different types of tumors and their core microorganisms. Although viral infections are known to cause tumors, studies on related viral diversity are scarce, and with the development of high-throughput sequencing technology, viral variety in tumor tissues should also be taken into account.

It is necessary to consider the tumor microbiota during tumor-related clinical treatment or scientific research. In addition, it is necessary to redefine the tumor microbiota. At this stage, research on the relationship between tumor therapies and tumor microbiota is still in its initial stage, and a lot of *in vivo* and *in vitro* studies are still needed. In this regard, an in-depth understanding of the distribution and function of the tumor microbiota is important to facilitate the understanding of oncogenic effects and to provide new therapeutic strategies for cancer(56). The tumor heterogeneity is a major issue especially in retrieving in a reliable and reproducible manner specific TME microbial markers. In addition, tumor tissue sampling methods are more or less limited, and microbial contamination can occur at any time between sampling and sequencing. Therefore, exogenous contamination must be eliminated through a strict sampling process.

Given the current paucity of clinical studies, studies at this stage have not been able to fully determine the relationship between tumor microbiota and tumor, and so many of the results are speculative. Therefore, further studies are needed to investigate how microorganisms enter tumor tissues, their relationship with tumor cells, and how to use them for cancer prevention, diagnosis, and prognosis.

## Author contributions

HZ, JC, and YL conceived and wrote the first draft of the manuscript. JC, HZ, YL, XC, and QL revised each part of the manuscript in detail. All authors contributed to the article and approved the submitted version.
